# Epstein–Barr Virus Monitoring after an Allogeneic Hematopoietic Stem Cell Transplant: Review of the Recent Data and Current Practices in Canada

**DOI:** 10.3390/curroncol31050211

**Published:** 2024-05-14

**Authors:** Claire Ratiu, Simon F. Dufresne, Stéphanie Thiant, Jean Roy

**Affiliations:** 1 Faculty of Medicine, Université de Montréal, Montréal, QC H3T 1J4, Canada; 2 Department of Microbiology, Infectious Diseases and Immunology, Faculty of Medicine, Université de Montréal, Montréal, QC H3T 1J4, Canada; 3 Division of Infectious Diseases and Clinical Microbiology, Department of Medicine, Maisonneuve-Rosemont Hospital, Montréal, QC H1T 2M4, Canada; 4 Centre de Recherche de l’Hôpital Maisonneuve-Rosemont, Montréal, QC H1T 2M4, Canada; 5 Division of Hematology, Oncology and Transplantation, Department of Medicine, Maisonneuve-Rosemont Hospital, 5415 de l’Assomption, Montréal, QC H1T 2M4, Canada

**Keywords:** rituximab, pre-emptive treatment, risk factors, Epstein–Barr virus, viral reactivation, post-transplant lymphoproliferative disorder, allogeneic stem cell transplant

## Abstract

Epstein–Barr virus-related post-transplantation lymphoproliferative disorder (EBV-PTLD) is a serious complication following hematopoietic stem cell transplantation (HSCT). A pre-emptive strategy using rituximab, which aims to manage patients early at the time of EBV reactivation to avoid PTLD, has been recommended by the most recent ECIL-6 guidelines in 2016. However, there is still a great heterogeneity of viral-load monitoring protocols, targeted patient populations, and pre-emptive treatment characteristics between centers, making precise EBV monitoring recommendations difficult. We conducted a literature review from the most recent publications between 1 January 2015 and 1 August 2023, to summarize the emerging data on EBV-PTLD prevention strategies in HSCT recipients, including the EBV-DNA threshold and use of rituximab. We also present the results of a survey of current practices carried out in 12 of the main HSCT centers across Canada. We confirm that pre-emptive rituximab remains an efficient strategy for EBV-PTLD prevention. However, there is an urgent need to perform prospective, randomized, multicentric trials with larger numbers of patients reflecting current practices to determine the best clinical conduct with regards to rituximab dosing, timing of treatment, and criteria to initiate treatments. Longer follow-ups will also be necessary to assess patients’ long-term outcomes.

## 1. Introduction

Hematopoietic stem cell transplant (HSCT) has revolutionized the treatment of patients with various hematological disorders, offering life-saving options that were previously unavailable. However, HSCT can also lead to significant complications, some of which are fatal, such as post-transplant lymphoproliferative disorder (PTLD). Its incidence ranges from 0.5% to 17% [1], depending on donor EBV serostatus, donor HLA match, conditioning regimen administered, and type of graft-versus-host disease (GvHD) prophylaxis used. PTLD has been associated with a mortality as high as 84% [2] in the absence of treatments and as low as 30% with appropriate therapy [1]. It is usually caused by the Epstein–Barr virus (EBV) [3], a widespread virus in adult populations worldwide, although some cases of EBV-negative PTLDs have also been reported [1,4]. After initial infection, EBV remains dormant in resting memory B cells [5,6]. However, in individuals with a decreased number and function of T lymphocytes (especially EBV-specific T cells), such as patients undergoing allogeneic stem cell transplantation or immunosuppression, EBV replication in B lymphocytes is left unchecked, potentially leading to B-cell transformation, uncontrolled proliferation, and PTLD. Virological monitoring of EBV reactivation has been proven to be a successful strategy for monitoring patients following HSCT and assessing the risk of PTLD development [7].

A pre-emptive strategy using anti-CD20 monoclonal antibodies is widely accepted and has been recommended by the most recent ECIL-6 guidelines published in 2016 [2]. This strategy aims to manage patients early enough (when DNA levels have reached a certain threshold) to avoid PTLD development. However, the question of when to start pre-emptive treatment remains highly controversial in the literature. An optimal threshold of EBV viral load would aim to avoid PTLD development in all patients at risk while minimizing unnecessary treatments and their associated side effects and costs. There is also wide heterogeneity around protocols for monitoring viral load. Many institutions use in-house developed assays, while several commercial kits are also available and utilized, which makes comparisons extremely difficult. Further complicating the assessment of strategies, published studies have reported on very different cohorts, with diverse patient populations, donor status, and conditioning regimens. This leaves important unanswered questions regarding the optimal strategies to prevent PTLD.

Considering that guideline recommendations are mostly based on heterogeneous clinical data dating back to several years and that many important questions might have evolved, we propose herein to re-examine the most recent literature. We also thought that portraying the landscape of current practices across Canada would be useful to Canadian clinicians by providing an opportunity for benchmarking, while also allowing for measuring adherence to the existing guidelines. We therefore undertook the present study, which comprises a comprehensive review of the recent knowledge about EBV epidemiology and management after HSCT and a survey on the current practices in Canada.

## 2. Methods

### 2.1. Review of Literature

A review of the literature was conducted in PubMed using the following research strategy: “Epstein–Barr virus” AND “stem cell transplant*” AND post-transplant* lymphoproliferative disorder”. Filters were applied for English language and publication date (articles published between 1 January 2015 and 1 August 2023). 

### 2.2. Survey on Current Practices in Canada

A survey was developed and distributed to all adult (*n* = 13) and pediatric transplant (n = 7) directors in Canada via the Cell Therapy Transplant Canada organization (https://www.cttcanada.org) accessed on 26 July 2023. 

## 3. Results

### 3.1. Literature Review

#### 3.1.1. Incidence of EBV-DNAemia after Allogeneic HSCT

A total of 26 articles describing the incidence of EBV reactivation were identified during our study period. The results of EBV reactivation after HSCT in both adults and children are shown in Table 1. Incidence remains highly variable, ranging from 30% to 68% in children [4,8,9,10,11] and 19% to 86% in adults [12,13,14,15,16,17,18,19,20,21,22,23,24,25,26,27,28,29,30,31] (excluding Marinho-Diaz et al., 2018, with an unrepresentative six adults studied). This variability reflects a high level of heterogeneity in the reported patient populations, including conditioning regimens, GvHD prophylaxis, donor type, or sensitivity of diagnostic tests. Similarly, the diagnosis criteria for EBV reactivation varies across studies. Overall reported incidences appear higher in the most recent literature, which could be owing to the wider use of EBV monitoring and increased use of alternative transplant grafts over time. The median time interval between HSCT and first EBV reactivation ranged from 31 [15] to 99 days [22], similar to previous studies. 

Several risk factors for EBV reactivation have been reported. Amongst them, some recipients’ characteristics, such as being a female recipient [11,21,32] and aged older than 40 years [19], were associated with EBV reactivation. A serostatus mismatch between an EBV-positive donor and an EBV-negative recipient or having an EBV-positive donor have also been described [9,11,14,15,27,31]. Some transplantation modalities contribute to an increased risk, such as the type of donor and the conditioning regimen. Male donors [19], matched unrelated donors [8,18,24,27,32], mismatched unrelated donors [21,24,27,32], and sibling donors [25,29] have been identified as risk factors. Only three studies have found that myeloablative and reduced intensity conditionings are associated with increased incidence [8,21,28]. On the other hand, the administration of anti-thymocyte globulin (ATG) [4,11,14,18,19,20,21,24,28,30,32,33], specifically horse ATG [9], is now a well-described risk factor. Interestingly, the administration of alemtuzumab seems to be associated with a lower incidence of reactivation, most likely due to its anti-B-cell properties [29,34]. In a study comparing ATG and alemtuzumab, the prevalence of EBV reactivation was 100% vs 58%, respectively [4], although not confirmed in another study [35]. These recent data underscore that all T-depletion strategies may not equally influence the risk of EBV reactivation, depending on their impact on decreasing B cells (the EBV reservoir) and on depleting T cells, more particularly EBV-specific memory T cells present in the graft. When comparing recipients of haploidentical donor (HID, no PTCy) versus recipients of haploidentical stem cell graft combined to an unrelated umbilical cord blood (haplo-cord), a similar EBV viremia was observed (6.3%). A recent meta-analysis showed that PTCy regimen versus ATG in allo-HSCT from unrelated donors was associated with a lower chance of developing EBV-related PTLD disease [36]. In the PTCy conditioning regimen, cyclophosphamide targets proliferating cells as NK and CD8+ T cells, but maintains CD4+ T cells in significant numbers compared to ATG, which could confer a clinical advantage to PTCy in maintaining control of EBV reactivation [37,38,39].

The long-term use of mycophenolate mofetil (MMF), which inhibits the reconstitution of NKp30 NK subsets, has also been correlated with a higher risk of EBV viremia [40]. After transplant, the development of some complications, as concomitant cytomegalovirus (CMV) infection with detectable DNA [19] or GvHD, whether acute or chronic, also increases the risk of EBV reactivation [9,14,19,21,24,31].

#### 3.1.2. Incidence of EBV-Related PTLD after Allogeneic HSCT

In the HSCT setting, PTLD is almost exclusively related to EBV infection, generally from a donor origin. The risk period for the occurrence of EBV-related PTLD is within the first 6 months post-transplant when T-cell immunity is not yet completely reconstituted [41]. The diagnosis of EBV-PTLD is based upon the identification of characteristic symptoms and/or signs, together with specialized tests, including quantitative the determination of EBV-DNAemia or detection of EBV in a specimen from the involved tissue, and imaging studies such as computed tomography (CT) or positron emission tomography CT (PET-CT). Definitive diagnosis needs to be confirmed by a histological examination of tissues suspected of being infected (biopsy) [41]. Different classification systems have been proposed, including the World Health Organization (WHO) classification of hematolymphoid tumors, recently revised in 2022 (5th edition) [42], but the post-HSCT PTLD subtype is rarely reported in publications.

The incidence of EBV-related PTLD is summarized in Table 1. Of note, PTLD subtypes are inconsistently reported and mostly not within the frame of the most recent WHO classification. Across studies, there is again a wide variability in the reported incidence, ranging from as low as 0% to 3.5% in children [4,8,9,10,11] and 0% to 14% in adults [12,13,14,15,16,17,18,19,20,21,22,23,24,25,26,27,28,29,30,31], falling within the range of previously reported incidences. Again, this variability most likely results from the heterogeneity of the transplant populations studied, GvHD prophylaxis, including the use of ATG or not, conditioning regimen administered, as well as donor EBV status. Similarly, larger studies, with >200 patients also report a high variability in EBV-PTLD incidence ranging between 0.2% and 14% [7,14,15,19,20,24,25,26,27,29,31]. Interestingly, Fujimoto et al. tailored a scoring system to rank patients from low (0–1) to very high risk (4–5) of developing PTLD based on the ATG dose used in the conditioning regimen (high = 2; low dose = 1), donor type (mismatched related donor = 1, unrelated donor = 1, and cord blood = 2), and a diagnosis of aplastic anemia (1 point). The probability of developing PTLD at 2 years post-HSCT ranged between 0.3% to 11.5% in low and very high-risk groups, respectively [43]. Recently, Che et al. validated Fujimoto’s PTLD scoring system and confirmed the acceptable discrimination of the system in a retrospective study of another cohort (n = 2148). They proposed to include additional predictors and showed that their new risk-group model (Lee’s LASSO score from 0 to 12) has better discrimination to identify high-risk patients [44]. Using these promising risk stratifications could help identify higher-risk patients who could benefit from more frequent or longer monitoring or universal prophylaxis.

#### 3.1.3. Optimal Biomarkers for PTLD Detection

EBV viral load (DNAemia) monitoring is a useful strategy for the management, diagnosis, and prediction of PTLD. At present, DNAemia remains the preferred biomarker, but important limitations and uncertainties remain. First, in the absence of a consensus, both commercial kits and different in-house assays with their own primers, probes, and choice of specimen are being used, thus inducing a very high level of heterogeneity across studies [45]. This lack of standardization has highlighted the importance and the need for internationally accepted guidelines. As an important step toward that goal, a WHO International Standard for EBV genome detection has been recently proposed [46].

Also, while the sensitivity and negative predictive values of EBV-DNAemia have been consistently high, positive predictive values are persistently low across the recent literature, with values ranging from 12.5% [47] to 73% [29]. In addition, both plasma and whole blood (WB) remain widely used, and optimal specimen remains a subject of active debate. Multiple studies have found WB to have a higher sensitivity than plasma [8,48,49]. It has further been reported that WB and peripheral blood mononuclear cells (PBMCs) have similar kinetics when evaluating EBV-DNAemia and that both specimens equally predict the risk of developing PTLD. These results are however challenged by Jennifer et al., who report that plasma EBV-DNAemia is a better predictor of PTLD compared to PBMCs [45,50]. Solano et al., however, report opposing results with the ineffectiveness of plasma EBV-DNA load to predict a high viral load or EBV-PTLD, even going so far as to question the clinical relevance of these tests [45].

Considering DNAemia limitations, alternative EBV-load monitoring methods have been proposed. Of interest, Fink et al. hypothesized that the number of infected B cells could be a more accurate biomarker [51]. As observed with cytomegalovirus, measuring patients’ immunological responses to EBV has also been explored as an alternative or complementary strategy to direct viral quantification. Importantly, there is a clear association between EBV-specific cytotoxic T lymphocytes (CTLs) and EBV reactivation [52,53]. Indeed, a study by Zhou et al. evaluated immune recovery in infected patients post-HSCT. High viral load was associated with a poor immune reconstitution of T lymphocytes and abnormally high levels of IL-10. They therefore suggested the dynamic monitoring of cytokines and T-cell reconstitution as a means of EBV infection monitoring to better predict PTLD development [54]. In a study by Althubaiti et al., very low T-cell counts (CD3+ <197, CD8+ <87) and a CD8+/CD20+ lymphocyte ratio < 1 were all found to be accurate cutoffs for predicting PTLD in the presence of EBV viremia, with the latter associated with negative and positive predictive values of 95% and 100%, respectively [10]. Monitoring T-cell functionality prior to HSCT has also been studied and suggested to better determine the individual risk of developing EBV reactivation [55]. These novel biomarkers show promise, but will need further clinical validation and optimization before incorporating them into clinical practice.

#### 3.1.4. Overall Efficacy of EBV-DNAemia-Based Pre-Emptive Strategy with Rituximab

The main objective of a pre-emptive strategy is to prevent the development of PTLD in patients with EBV-DNAemia after allogeneic HSCT. The most commonly used pre-emptive strategy associates the use of rituximab (RTX) with a reduction in immunosuppression whenever possible. After the first dose of RTX, B-cell counts start to decrease, thus limiting the ability of EBV to replicate. 

Table 2 describes the overall efficacy of RTX-based pre-emptive treatment for EBV reactivation and EBV-related PTLD as reported in eight recent studies. Of note, all were monocentric retrospective cohort studies. The overall efficacity for EBV clearance varied between 85% [28] and 100% [12] for patients with EBV reactivation and between 65% and 100% for EBV-related PTLD. In the single largest cohort of adult patients reported to date (n = 107 treated patients), EBV clearance was achieved in 95% [56]. In pediatric patients, the efficacy of pre-emptive strategies for EBV clearance was similar, ranging from 89% [57] (HID transplant) to 100% [58]. These studies all conclude that pre-emptive therapy of EBV reactivation post-HSCT is efficient in the control of EBV reactivation and may effectively prevent PTLD. Collectively, these recent data expand the body of literature supporting current clinical practice guidelines. However, the quality of evidence remains relatively low, and prospective controlled studies are still missing. 

It should also be noted that pre-emptive protocols present variability in terms of the EBV-DNAemia threshold chosen to start RTX, as well as in terms of the number of doses administered (from 1 to 8 doses). There is also heterogeneity in types of donors (HID vs matched donor), donor serologic status, conditioning regimen, and GvHD prophylaxis used. All these factors were shown to impact on EBV reactivation post-HSCT (Table 1). All studies used a dose of RTX of 375 mg/m^2^, except one [17] that tested 100 mg/m^2^. Low-dose RTX could therefore be useful in treating patients as efficiently, while keeping toxicity low. However, the official ECIL-6 guidelines recommend a dose of 375 mg/m^2^ once weekly until EBV-DNAemia negativity is reached. ECIL-6 also recommends assessing patients’ immune function and EBV-DNAemia kinetics prior to determine the number of doses, which typically ranges from one to four [2]. One study has found that higher EBV viral loads might require more than a single dose of RTX [59].

#### 3.1.5. Optimal Threshold for EBV-DNAemia-Driven Therapy

The optimal threshold for initiating pre-emptive therapy is perhaps the most challenging aspect of PTLD management. Ideally, patients with a low risk of PTLD should not be unnecessarily treated, thus avoiding drug-induced toxicity. A high threshold would miss some patients who will eventually develop PTLD and would imply treating more patients therapeutically rather than pre-emptively. Conversely, a lower threshold would imply treating more patients, some of them unnecessarily, thus exposing them to unwanted side effects. The ECIL-6 guidelines do not advise on a specific threshold, and rather suggest following locally defined cutoffs. With many centers having adopted a DNAemia-based pre-emptive strategy, the issue of an optimal threshold is being actively investigated. 

First, recent literature suggests that a static EBV viral load has been shown to be a better and simpler biomarker than kinetics of EBV-DNA, making it the biomarker of choice to predict the risk of developing PTLD [15,60]. Using static DNAemia measured by an in-house assay and the RealStar assay, Kalra et al. attempted to evaluate the optimal threshold for pre-emptive RTX therapy and suggested the use of a target of 100,000 to 500,000 IU/mL in WB. With a threshold of 100,000 IU/mL, there would be no fatal PTLD, but 20% of patients would be treated unnecessarily. With a higher threshold of 500,000 IU/mL, 0.3% of patients would die from PTLD, but only 3.9% of patients would be treated unnecessarily [15]. Another study, focusing on high-risk patients, found that a viral load above 10,000 IU/mL in WB was the strongest predictor of developing PTLD, with a sensitivity of 94.8% and a specificity of 94.4%, supporting the use of a lower threshold between 5000 and 10,000 IU/mL [28]. Worsening of clinical manifestations or a significant increase in viremia is also considered as a signal to start pre-emptive treatment [2,11]. Raberahona et al. showed that only treated patients with an EBV viral load greater than 50,000 IU/mL had better overall survival at 3 years post-treatment compared to untreated patients [14]. 

As mentioned previously, the absence of a consensus on the EBV level threshold is also due to the lack of PCR method standardization, the type of units expressed, and the different sample materials that can be used (WB, plasma, or PBMCs) [2,61]. There are no data to support a preference for WB, plasma, or serum; according to the ECIL-6 guidelines, all are appropriate specimens for monitoring EBV DNAemia. When plasma is used, the threshold of 1000 copies/mL (defined as persistent or on two consecutive occasions) was mostly used [17,34,62] or the corresponding 1000 copies/10^6^ in PBMCs [57]. When WB is used, this value can range from 40,000 copies/mL [4,58], knowing that EBV load in WB is considered as 10- to 100-fold higher than in plasma samples [63]. Stocker et al. proposed a threshold from 5000 IU/mL in WB, whereas Lindsay et al. treated patient with 1000 to 100,000 IU/mL in plasma. If we consider that the conversion factor for EBV is often 1 IU/mL = 1 copy/mL, these two studies started RTX prophylaxis in the same range than previously described. The conversion factor is however variable from a manufacturer to another [64], which makes the comparison of studies difficult. Similar to previously published 2016 ECIL-6 guidelines, no specific threshold of DNAemia can currently be recommended for the initiation of pre-emptive therapy based on the most recent data.

#### 3.1.6. Efficacy of Universal Primary Prophylaxis Strategy

The objective of a universal prophylaxis strategy is to prevent EBV reactivation in most (ideally all) patients using an anti-CD20 monoclonal antibody prior to HSCT. Since 2015, only two studies have used RTX alone before transplantation to evaluate the incidence of EBV reactivation. Patel et al. reported a single-center retrospective analysis comparing 43 patients who received one RTX prophylactic dose of 375 mg/m^2^ before HLA identical allogeneic HSCT with 43 patients who did not in the same HSCT setting [65]. They observed no EBV reactivation at day + 180 and no EBV-PTLD occurrence at one year in the 43 patients with prophylactic RTX versus 53% of EBV reactivation at day + 180 and 14% of EBV-PTLD in the untreated group. There was no difference in the incidence of GvHD and infection episodes between both groups [65]. These remarkable results were confirmed in a prospective cohort study of haplo-cord transplantation, where 51 patients received pre-transplant RTX and 147 patients did not. EBV reactivation occurred in only 2% of patients who received one prophylactic dose (375 mg/m^2^) of RTX vs 13% in those untreated [66] on day + 180 post-transplant. No PTLD developed in the RTX treated group versus 8% in untreated patients. 

RTX can have effects on acquired immunity. It is known to induce a prolonged reduction of immunoglobulin levels (up to 12 months) and delays in B-cell reconstitution, leading to an increased incidence of sepsis including fatal infections [67]. However, while another study reported RTX to be linked to lymphopenia, it did not confirm an increased incidence of overall infections between RTX treated patients and controls 2 years after HSCT [56]. Interestingly, RTX was also found to have a protective effect on both acute [68] and chronic GvHD [69,70] when used for prevention of EBV reactivation. RTX prophylactic strategy may represent a viable option, but additional studies are still needed. 

#### 3.1.7. Alternative Therapies for EBV-DNAemia

First-line therapy of patients who develop EBV reactivation after HSCT relies on a reduction in immunosuppression whenever possible and the administration of an anti-CD20 monoclonal antibody, such as RTX [2]. Reduction in immunosuppression is defined as a sustained decrease of at least 20% of the daily dose of immunosuppressive drugs with the exception of low-dose corticosteroid therapy [2,71]. It is noteworthy that this reduction remains unstandardized, therefore widely variable from one physician and center to another. When RTX therapy fails to achieve viral clearance, donor lymphocyte infusions (DLIs) or EBV-CTLs can be used as second-line therapy [2,53]. In their most recent publication, Jiang et al. infused EBV-CTLs from third-party donors and demonstrated complete EBV clearance in 94% of patients, resulting in specific immune reconstitution and a low incidence of disease recurrence [72]. Another recent study reported the use of ganciclovir, foscarnet, and intravenous immunoglobulins in addition to reduced immunosuppression for EBV-positive recipients, with a low incidence of PTLD of only 3.3% [52]. Our review of the recent literature does not support any change in ECIL-6 recommendations regarding alternative therapies. 

### 3.2. Current Practices in Canadian Transplant Centers

A total of eight centers completed the adult survey (Princess Margaret Cancer Center, Toronto; Saskatoon Cancer Center, Saskatoon; Alberta Blood and Marrow Transplant Program, Calgary; Hôpital Maisonneuve-Rosemont, Montréal; British Columbia *Leukemia* and Bone Marrow *Transplant Program*, Vancouver; Ottawa Hospital, Ottawa; Cancer Care Manitoba, Winnipeg; and Centre hospitalier universitaire (CHU) de Québec, Quebec City) and four pediatric centers (Alberta Children’s Hospital, Calgary; Cancer Care Manitoba, Winnipeg; Sick Kids Hospital, Toronto; and CHU Sainte-Justine, Montréal) completed the survey. If centers did not perform systematic EBV monitoring, the survey was nevertheless completed to establish their current clinical practice. The results are summarized in Table 3.

Surprisingly, only 37.5% of adult centers perform systematic EBV monitoring vs. 100% of pediatric centers in Canada. In adult centers, 37.5% consider only high-risk patients to be suitable monitoring candidates. In these centers, high-risk profiles were based on ATG administration for GvHD prophylaxis, patients undergoing HID transplants, patients with serology mismatch, or patients developing acute GvHD.

In both adult and pediatric centers, a quantitative EBV PCR is used for monitoring, and this assay is performed locally. WB or plasma specimens are equally used in pediatric centers. A total of 50% of adult centers declared using WB, 25% plasma, and 25% did not answer. Among adult centers, one quarter used commercially available kits, while another quarter used laboratory-developed tests. Half of them were uncertain about the assay employed. Similarly, half of the pediatric centers used a commercially available test kit, while the other half used a laboratory-developed test.

Weekly monitoring was performed in a majority of centers (62.5% in adult vs. 75% in pediatric centers). Regarding adult centers, frequency adjustments were implemented in response to a rapid increase in viremia, after the tapering of immunosuppressants (ISs) and after 3 months or when patient visits were less frequent. Only one center did not adjust the monitoring frequency. In contrast, half of the pediatric centers did not modify their monitoring frequency or rarely, while the other half did, as patients moved away from their transplant date and providing there were no concerns the virus was causing clinical or laboratory problems. 

Among the centers that perform monitoring, 80% and 75% adult and pediatric centers, respectively, have adopted a pre-emptive approach. EBV viremia thresholds for initiating this strategy varied dramatically: 12% and 50% of adult and pediatric centers, respectively, relied on the physician’s judgment, while the remaining centers established their own threshold ranging from 10,000 to 300,000 copies/mL for the WB test and 5000 copies/mL for plasma analysis. The two other pediatric centers and single adult center using plasma tests did not comment on their thresholds. Treatment approaches included a reduction in IS, administration of RTX, or a combination of both as first-line treatment across all centers. Those who favored IS reduction as first line would start RTX as second line and vice versa. The infusion of donor lymphocytes was also considered as second-line treatment in one adult center, whereas antivirals and CTLs were reserved as third-line treatment alternatives in some pediatric and adult centers. 

Pre-emptive surveillance was continued for a minimum of 3 months in four adult centers. One center evaluated the magnitude of EBV-DNAemia along with the presence of EBV-related symptoms and response to RTX before deciding to continue surveillance, while another maintained weekly surveillance until IS was discontinued or after a one-month threshold, whichever came later. Concerning pediatric centers, all centers use different thresholds for the discontinuation of surveillance: one continues until 2 years, another continues for 2–3 months, and another continues until viremia clearance on at least two measurements. Finally, only one pediatric center performs a systematic surveillance strategy for late-onset PTLD, which consists of viral-load monitoring until 2 years post-HSCT, whereas no adult center does it. 

## 4. Conclusions and Future Directions

We observed through this literature review that the management of EBV reactivation following HSCT varies drastically across centers in Canada and worldwide, sometimes relying solely on physicians’ clinical judgments. Although the ECIL-6 recommendations for the diagnosis of EBV DNAemia and pre-emptive therapy for EBV disease remain accurate, the management of EBV after allogeneic HSCT still represents a complex clinical landscape, further complicated by current changes in the practice with the increasing use of HIDs and matched or mismatched unrelated donors with the use of post-transplant cyclophosphamide for GvHD prophylaxis [73]. The incidence and kinetics of EBV-DNAemia and disease in these patients remain to be clarified. Following our recent literature review, we can summarize the most important observed issues at three levels: (1) Persistent inconsistency and wide heterogeneity in published cohorts that contribute to the variability in reported incidences and risk factors in the literature, (2) the absence of universal standards for EBV-DNAemia assays that poses a problem for comparing studies and determining a clear threshold for initiating pre-emptive treatment, and (3) the lack of prospective controlled trials evaluating and comparing universal prophylaxis and pre-emptive approaches.

Future research and collaborative efforts should therefore focus on carrying out prospective multicentric trials with larger numbers of homogeneous transplant recipients reflecting current practices with long-term follow-up to (1) determine the incidence and risks factors for each type of patient and transplant modality, (2) confirm the superiority of the pre-emptive approach over other strategies and determine its optimal parameters with regards to biomarker utilization and RTX administration modalities, and (3) develop and validate risk scores and decision-making algorithms personalized and risk-tailored for each patient. Finally, it is imperative to standardize and define protocols for the monitoring of EBV reactivation to ensure the adequate surveillance of all patients across different HSCT centers in Canada and elsewhere. Alternative immunity-directed monitoring tools, as well as novel therapeutics, also remain of great interest to this field and will require more attention in the future.

## Figures and Tables

**Table 1:** Epidemiology of EBV reactivation and PTLD in studies published between 2015 and 2023.

N Patients Studied	Type of Transplant	Conditioning	GvHD Prophylaxis	% of EBV Reactivation	% PTLD	Median Day of EBV-DNA Detection	Identified EBV-DNAemia Risk Factors after Multivariate Analysis	References
186 Adults	MUD MSD MMUD MMRD	MAC, RIC	CyA +/− MTX or MMF	48% ≥ 500 genomes/mL 18% ≥ 20,000 genomes/mL	4.3%	N/A	N/A	Burns [12] (2016)
28 Pediatric	MUD MRD	MAC, RIC	CyA, CyA + MTX Rabbit ATG (2–5 mg/kg)	46.4%	N/A	47	MUD, MAC	Chiereghin [8] (2016)
30 Adults Adolescents > 14	MUD MSD HID	MAC	CyA + MTX CyA + MTX + MMF Rabbit ATG (10 mg/kg)	47%	6.7%	31	N/A	Fu [13] (2016)
332 Adults	N/A	TBI, Flu, Other (NP)	ATG (NP)	69.6% ≥ 1000 copies/mL	N/A	98	D-EBV status, ATG, Flu, TBI MUD, GvHD	Raberahona [14] (2016)
182 Pediatric	MUD HID	MAC, RIC, NMA	Rabbit ATG (5 mg/kg) or horse ATG (100 mg/kg), T depletion, other	33%	0.5%	94.5	Acute GvHD grade II to IV, Horse ATG EBV serostatus D+ R−,	Laberko [9] (2017)
306 Adults	MUD MSD MMUD CB	MAC	CyA, MTX	82%	14%	33	EBV serostatus D+ R−	Kalra [15] (2018)
50 Adults	HID	RIC	PTCy CyA Rabbit ATG (5 mg/kg)	64%	8%	N/A	N/A	Law [16] (2018)
15 Adults (6) Pediatric (9)	MRD MMUD CB	MAC, RIC	ATG (NP) Tacrolimus + MTX CyA + MTX, Tacrolimus	100%	50% (Adult) 25% (Pediatric)	N/A	N/A	Marinho-Dias [32] (2018)
199 Adults	MUD MSD, HID, CB	MAC, RIC	ATG (NP)	50%	0.5%	N/A	N/A	Delapierre [17] (2019)
266 Pediatric	MUD MRD	MAC, NMA	CyA + MTX, CyA + other MMF, ATG (NP) Alemtuzumab	30%	3%	N/A	N/A	Althubaiti [10] (2019)
123 Adults	MUD MRD	MAC, RIC, NMA	Rabbit ATG (4 mg/kg) Tacrolimus + MMF Tacrolimus + MTX	24%	N/A	N/A	MUD with ATG	Figgins [18] (2019)
200 Adults	MRD HID	MAC	Rabbit ATG (2.5 mg/kg), MTX + MMF + CyA	44%	11.9%	42	For MRD: ATG, male D, CMV-DNAemia, For HID: donor age > 40, CR at transplant, CMV-DNAemia,	Gao [19] (2019)
408 Adults	HID	MAC, RIC	MTX+ CyA + MMF Rabbit ATG (7.5 or 10 mg/kg)	20.7% (ATG 7.5 mg) 40% (ATG 10 mg)	N/A	N/A	ATG dose	Lin [20] (2019)
40 Adults	MRD MMUD CB	MAC, RIC	ATG (NP)/none	70%	0%	N/A	Female R, UD, HLA-MM, PBSCs, MAC, ATG, acute GvHD	Marinho-Dias [21] (2019)
63 Adults	HID	MAC, RIC	PTCy/Tacrolimus/MMF	28.5%	0%	99	N/A	Mohyuddin [22] (2019)
186 Adults	MRD MUD HID	MAC, RIC	CyA + MTX CyA + MTX + MMF Rabbit ATG (6–10 mg/kg)	18.8%	0%	53	BM graft	Wang [23] (2019)
890 Adults	MRD HID CB?	MAC, RIC	CyA + MTX ± MMF + ATG (NP)	19.7%	0.2%	57	ATG, HLA-MM, chronic GvHD	Ru [24] (2020)
270 Adults	MRD MUD MMUD HID	RIC, TBI	Rabbit ATG (4.5 mg/kg), PTCy, CyA	63.7%	12%	68	MRD	Salas [25] (2020)
156 Pediatric	MRD MMRD CB	MAC, Other	ATG (NP) Alemtuzumab, CyA, MTX, MMF, Tacrolimus	42.3%	3.2%	N/A	R EBV+, D EBV+, ATG, female R	Enok Bonong [11] (2021)
296 Adults	MUD MMUD HID	MAC	CyA + MTX + MMF Rabbit ATG (10 mg/kg)	42.6%	0.67%	48	N/A	Ke [26] (2021)
382 Adults	MRD MMRD MUD	MAC, RIC	CyA + MMF, CyA + MTX ATG (NP)	56.5%	1.3%	35	HLA-MM, TBI, UD, EBV IgG donor serology, CyA/MTX and ATG use of GvHD prophylaxis	Macy [27] (2021)
405 Adults	MRD, MUD, HID, CB	MAC, RIC	ATG (4.5 mg/kg), CyA + MTX	54.8%	5.4%	N/A	ATG	Lindsay [28] (2021)
515 Adults	MRD MUD MMUD	RIC, MAC	Alemtuzumab CyA	35.8%	3.9%	89.5	MRD	Marzolini [29] (2021)
61 Adults	HID	MAC, RIC	PTCy +/− Rabbit ATG (4.5 mg/kg) CyA + MMF Sirolimus + MMF	55.8% (ATG) vs. 12.5% (no ATG)	N/A	N/A	ATG	Chen [30] (2022)
56 Pediatric	MRD MUD MMR MMUD	MAC, MIC, RIC	ATG (NP) Alemtuzumab	67.9%	1.8%	40	R EBV+, ATG	Kania [4] (2022)
1184 Adults	MRD MUD MSD	RIC, Other	ATG (4.5 mg/kg) + MTX + CyA	86%	9%	35	For PTLD: EBV D+/R−, TBI, non-MRD (sibling)	Kinzel [31] (2022)

ATG: anti-thymocyte globulin; BEAM: carmustine, etoposide, arabinoside-C, melphalan; Bu: busulfan; BM: bone marrow; CB: cord blood; Cy: cyclophosphamide; CyA: cyclosporine A; D: donor; EBV: Epstein–Barr virus; Flu: fludarabine; GvHD: graft-versus-host disease; HID: haploidentical donor; MAC: myeloblative conditioning; MIC: minimal-intensity conditioning; Mel: melphalan; MMF: mycophenolate mofetil; MRD: matched related donor; MSD: matched sibling donor; MMRD: mismatched related donor; MMUD: mismatched unrelated donor; MUD: matched unrelated donor; MM: mismatched UD: unrelated donor; MTX: methotrexate; N/A: Not applicable; NMA: non-myeloablative; NP: not provided; PBSC: peripheral blood stem cell; R: recipient; RIC: reduced intensity conditioning; TBI: total body irradiation.

**Table 2:** Rituximab-based pre-emptive treatment for EBV reactivation and EBV-related PTLD–review from 2015 to 2023.

N Patients Treated with Rituximab	Donor Type	EBV-DNAemia Threshold	Rituximab Dosage and # of Doses	% of Patients with Clearance	Time of Clearance Assessment	% Relapse of EBV	% PTLD	References
30 (EBV) 8 (PTLD) Adults	MSD MUD MMRD MMUD	>20,000 copies/mL	375 mg/m^2^ up to 4 weekly doses	100 (EBV) 63 (PTLD)	UK	0	N/A	Burns [12] (2016)
19 Pediatric	HID	>1000 copies/10^6^ PBMCs	375 mg/m^2^ single dose	89 (1 dose only)	UK	0	0	Kobayashi [57] (2017)
61 Adults	MRD MUD MMRD MMUD	Copies > 2.5 limit of detection OR sustained rising levels of viral load	375 mg/m^2^ weekly until viremia clearance	52 (1 dose) 97 (1–4 doses)	Median of 5 days post-RTX Median (range) of 9 days (1–41) post-RTX initiation	1.4	1.4	Jain [59] (2017)
28 (EBV) 6 (PTLD) Adult and pediatric patients	RD UD	1000 gE/mL × 2 occasions OR 10,000 gE/mL in one sample	375 mg/m^2^ weekly until viremia <1000 gE/mL and resolution of clinical signs	89 (1–6 doses) 83 * (4–6 doses)	UK	12	18	Kinch [34] (2018)
16 Adults	MSD MUD HID CB	>1000 IU/mL OR without possibility of IS reduction	100 mg/m^2^ weekly until viremia decreased of 1 log10 and below 1000 IU/mL	93 (1–4 doses)	After 4 doses	N/A	6.3	Delapierre [17] (2019)
19 Pediatric	RD UD HID	40,000 copies/mL	375 mg/m^2^ single dose	100 (1 dose only)	Median (range) of 9 days (3–20) from RTX	0	0	Kim [58] (2019)
107 Adults	MRD MUD HID	2 consecutive viral loads in whole blood >5000 IU/mL	375 mg/m^2^ weekly until viremia clearance	95 (1–8 doses)	UK	N/A	5	Stocker [56] (2020)
20 Adults	MRD UD CB HID	11 treated >1000–10,000 IU/mL 3 treated >10,000–100,000 IU/mL 6 treated >100,000 IU/mL	375 mg/m^2^ weekly until viremia clearance	85 (1–4 doses)	UK	N/A	15	Lindsay [28] (2021)

* 3 EBV-PTLD patients received concomitant chemotherapy (COP: cyclophosphamide, vincristine, and prednisone (n = 1) and cyclophosphamide (n = 2)). All were retrospective cohort studies. CB: cord blood; EBV: Epstein–Barr virus; gE: genome equivalent; IUs: international units; HID: haploidentical donor; MRD: matched related donor; MMRD: mismatched related donor; MSD: matched sibling donor; MMUD: mismatched unrelated donor; MUD: matched unrelated donor; N/A: Not applicable; RD: related donor; UD: unrelated donor; PBMC: peripheral blood mononuclear cell.

**Table 3:** Canadian survey responses (n = 12 centers).

Questions	Answers	Pediatric Centers n = 4	Adult Centers n = 8
1. Is there a systematic EBV monitoring strategy at your center?	Yes No	4 NA	6 2
2. What ASCT patients are considered for systematic EBV monitoring?	All High-risk only No answer	4 NA NA	3 3 2
2a. If you have selected “only patients at high risk” at the previous question, please specify.	AC 1: EBV serology mismatch/Use of ATG for GvHD prophylaxis/use of Alemtuzumab/MUD/MMUD/HID/Grade III-IV acute GvHD/Steroid-refractory acute GvHD. AC 2: EBV serology mismatch/Use of ATG for GvHD prophylaxis/MUD/MMUD/HID/CB/Any acute GvHD treated with oral prednisone or IV solumedrol. AC 3: Use of ATG for GvHD prophylaxis/HID/CB/any acute GvHD		
3. Where is EBV detection assay performed?	At our center No answer	4 NA	6 2
4. What technique is used for EBV monitoring?	Quantitative PCR Quantitative/Qualitative PCR No answer	3 1 NA	6 NA 2
5. What specimen is utilized?	Whole blood Plasma No answer	2 2 NA	4 2 2
6. What type of assay is used?	Commercial assay LDT No answer	2 2 NA	2 2 4
7. When is EBV monitoring ended after transplantation?	D + 100–120 D + 180 2 years D + 180/when IS are stopped D + 100–120/extend if prolonged IS When IS are stopped No answer	1 1 1 1 NA NA NA	1 1 NA 1 1 2 2
8. At what frequency is EBV monitored?	Weekly Every 2 weeks Weekly until D + 100 then less frequently No answer	3 NA 1 NA	5 1 NA 2
9. Do you modify this frequency in certain circumstances?	Yes No Rarely No answer	2 1 1 NA	5 1 NA 2
9a. If you have selected “yes” at the previous question, please specify.	PC 1: Spread out as the patient further from HSCT and less seen, and there is no concern with clinical or laboratory EBV related problems AC1/2: Rising EBV PCR AC3: Q2 weeks once started tapering of IS AC 4: When >3 months and on prolonged IS, may be less frequent if not being seen in clinic weekly AC 5: If positive result weekly analysis can be used instead every 2 weeks		
10. Do you perform preemptive treatment strategy for EBV reactivation/PTLD?	Yes No No answer	3 1 NA	6 NA 2
11. Regarding preemptive strategy, what threshold is used to start therapy?	Specific number of copies/mL No fixed threshold, physician decision No answer	1 2 1	5 1 2
11a. For “specific number of copies/mL”, please specify the number.	PC 1: 10,000 AC 1: >300,000 to treat; >30,000 PTLD investigation AC 2: 300,000 without symptoms, 30,000 with symptoms (fever, rash, lymphocytosis, lymph node enlargement) AC3: 5000 AC4/5: 10,000		
12. What is your 1st line of therapy for asymptomatic EBV-DNAemia?	Reduction of IS Rituximab Rituximab + Reduction of IS No answer	1 1 1 1	2 NA 4 2
13. What is your 2nd line of therapy for refractory asymptomatic EBV-DNAemia (no PTLD)?	Reduction of IS Rituximab Rituximab + Reduction of IS Further IS reduction Donor lymphocyte infusion No answer	1 1 NA 1 NA NA	NA 2 1 NA 1 4
14. What is your 3rd line of therapy for refractory asymptomatic EBV-DNAemia (no PTLD)?	Anti-virus specific T cells No answer No patient has really made it to third line	1 3 NA	2 5 1
15. How long after an intervention for EBV-DNAemia do you continue surveillance?	2–3 months 3–6 months ~3 Months At least 3 months Until 2 years Clearance of virus (2 measurements) Depends on outcome, severity, response Weekly until IS discontinued or 4 weeks No answer	1 NA NA NA 1 1 NA NA 1	NA 1 1 2 NA NA 1 1 2
16. Do you have a systematic surveillance strategy for late-onset PTLD?	Yes No No answer	1 3 NA	2 4 2
16a. If you have selected “yes” at the previous question, please specify.	PC 1: Virus Monitoring until 2 years post ASCT AC 1: Patients who had previous reactivation can be monitored longer AC 2: Weekly for the first 3 months or if GvHD. Bi-weekly once tapering IS and not previous activation. Stop once IS off		

ASCT: allogeneic stem cell transplant; CB: cord blood; D: day; GvHD: graft-versus-host disease; IS: immunosuppressant; MUD: matched unrelated donor; MMUD: mismatched unrelated donor; HID: haploidentical donor; LDT: laboratory-developed test; PC: pediatric center; AC: adult center.

## References

[1] Al Hamed R., Bazarbachi A.H., Mohty M. (2020). Epstein-Barr virus-related post-transplant lymphoproliferative disease (EBV-PTLD) in the setting of allogeneic stem cell transplantation: A comprehensive review from pathogenesis to forthcoming treatment modalities. Bone Marrow Transpl..

[2] Styczynski J., van der Velden W., Fox C.P., Engelhard D., de la Camara R., Cordonnier C., Ljungman P. (2016). Management of Epstein-Barr Virus infections and post-transplant lymphoproliferative disorders in patients after allogeneic hematopoietic stem cell transplantation: Sixth European Conference on Infections in Leukemia (ECIL-6) guidelines. Haematologica.

[3] Pegoraro F., Favre C. (2021). Post-transplantation lymphoproliferative disorder after haematopoietic stem cell transplantation. Ann. Hematol..

[4] Kania S.P., Silva J.M.F., Charles O.J., Booth J., Cheung S.Y.A., Yates J.W.T., Worth A., Breuer J., Klein N., Amrolia P.J. (2022). Epstein-Barr Virus Reactivation After Paediatric Haematopoietic Stem Cell Transplantation: Risk Factors and Sensitivity Analysis of Mathematical Model. Front. Immunol..

[5] Zanelli M., Sanguedolce F., Palicelli A., Zizzo M., Martino G., Caprera C., Fragliasso V., Soriano A., Valle L., Ricci S. (2021). EBV-Driven Lymphoproliferative Disorders and Lymphomas of the Gastrointestinal Tract: A Spectrum of Entities with a Common Denominator (Part 1). Cancers.

[6] Al Tabaa Y., Tuaillon E., Bollore K., Foulongne V., Petitjean G., Seigneurin J.-M., Duperray C., Desgranges C., Vendrell J.-P. (2009). Functional Epstein-Barr virus reservoir in plasma cells derived from infected peripheral blood memory B cells. Blood.

[7] Lindsay J., Yong M.K., Greenwood M., Kong D.C.M., Chen S.C.A., Rawlinson W., Slavin M. (2020). Epstein-Barr virus related post-transplant lymphoproliferative disorder prevention strategies in allogeneic hematopoietic stem cell transplantation. Rev. Med. Virol..

[8] Chiereghin A., Prete A., Belotti T., Gibertoni D., Piccirilli G., Gabrielli L., Pession A., Lazzarotto T. (2016). Prospective Epstein-Barr virus-related post-transplant lymphoproliferative disorder prevention program in pediatric allogeneic hematopoietic stem cell transplant: Virological monitoring and first-line treatment. Transpl. Infect. Dis..

[9] Laberko A., Bogoyavlenskaya A., Shelikhova L., Shekhovtsova Z., Balashov D., Voronin K., Kurnikova E., Boyakova E., Raykina E., Brilliantova V. (2017). Risk Factors for and the Clinical Impact of Cytomegalovirus and Epstein-Barr Virus Infections in Pediatric Recipients of TCR-α/β- and CD19-Depleted Grafts. Biol. Blood Marrow Transpl..

[10] Althubaiti S., Ali S., Renzi S., Krueger J., Chiang K.-Y., Schechter T., Punnett A., Ali M. (2019). Lymphocyte subset at time of Epstein-Barr viremia post-allogeneic hematopoietic stem cell transplantation in children may predict development of post-transplant lymphoproliferative disease: CD8:CD20 ratio as a sensitive predictor. Pediatr. Transpl..

[11] Enok Bonong P.R., Buteau C., Duval M., Lacroix J., Laporte L., Tucci M., Robitaille N., Spinella P.C., Cuvelier G.D.E., Lewis V. (2021). Risk factors for post-transplant Epstein-Barr virus events in pediatric recipients of hematopoietic stem cell transplants. Pediatr. Transpl..

[12] Burns D.M., Rana S., Martin E., Nagra S., Ward J., Osman H., Bell A.I., Moss P., Russell N.H., Craddock C.F. (2016). Greatly reduced risk of EBV reactivation in rituximab-experienced recipients of alemtuzumab-conditioned allogeneic HSCT. Bone Marrow Transpl..

[13] Fu L., Wang J., Wei N., Wu L., Wang Y., Huang W., Zhang J., Liu J., Wang Z. (2016). Allogeneic hematopoietic stem-cell transplantation for adult and adolescent hemophagocytic lymphohistiocytosis: A single center analysis. Int. J. Hematol..

[14] Raberahona M., Wackenheim C., Germi R., Carré M., Bulabois C.-E., Thiébaut A., Lupo J., Semenova T., Cahn J.-Y., Morand P. (2016). Dynamics of Epstein-Barr viral load after hematopoietic stem cell transplantation and effect of preemptive rituximab therapy. Transpl. Infect. Dis..

[15] Kalra A., Roessner C., Jupp J., Williamson T., Tellier R., Chaudhry A., Khan F., Taparia M., Jimenez-Zepeda V.H., Stewart D.A. (2018). Epstein-barr virus DNAemia monitoring for the management of post-transplant lymphoproliferative disorder. Cytotherapy.

[16] Law A.D., Salas M.Q., Lam W., Michelis F.V., Thyagu S., Kim D.D.H., Lipton J.H., Kumar R., Messner H., Viswabandya A. (2018). Reduced-Intensity Conditioning and Dual T Lymphocyte Suppression with Antithymocyte Globulin and Post-Transplant Cyclophosphamide as Graft-versus-Host Disease Prophylaxis in Haploidentical Hematopoietic Stem Cell Transplants for Hematological Malignancies. Biol. Blood Marrow Transpl..

[17] Delapierre B., Reman O., Dina J., Breuil C., Bellal M., Johnson-Ansah H., Gac A.C., Damaj G., Chantepie S. (2019). Low dose Rituximab for pre-emptive treatment of Epstein Barr virus reactivation after allogenic hematopoietic stem cell transplantation. Curr. Res. Transl. Med..

[18] Figgins B., Hammerstrom A., Ariza-Heredia E., Oran B., Milton D.R., Yeh J. (2019). Characterization of Viral Infections after Antithymocyte Globulin-Based Conditioning in Adults Undergoing Allogeneic Hematopoietic Stem Cell Transplantation. Biol. Blood Marrow Transpl..

[19] Gao X.-N., Lin J., Wang L.-J., Li F., Li H.-H., Wang S.-H., Huang W.-R., Gao C.-J., Yu L., Liu D.-H. (2019). Risk factors and clinical outcomes of Epstein-Barr virus DNAemia and post-transplant lymphoproliferative disorders after haploidentical and matched-sibling PBSCT in patients with hematologic malignancies. Ann. Hematol..

[20] Lin R., Wang Y., Huang F., Fan Z., Zhang S., Yang T., Xu Y., Xu N., Xuan L., Ye J. (2019). Two dose levels of rabbit antithymocyte globulin as graft-versus-host disease prophylaxis in haploidentical stem cell transplantation: A multicenter randomized study. BMC Med..

[21] Marinho-Dias J., Baldaque I., Pinho-Vaz C., Leite L., Branca R., Campilho F., Campos A., Medeiros R., Sousa H. (2019). Association of Epstein-Barr virus infection with allogeneic hematopoietic stem cell transplantation in patients in Portugal. Mol. Med. Rep..

[22] Mohyuddin G.R., Roller J., Shune L., Lin T., Dias A., Ganguly S., Abhyankar S., McGuirk J., Singh A. (2019). Epstein-Barr viremia and post-transplant lymphoproliferative disorders in patients undergoing haploidentical stem cell transplantation with post-transplant cyclophosphamide. Hematol. Oncol. Stem Cell Ther..

[23] Wang H., Zhang T.-T., Qi J.-Q., Chu T.-T., Miao M., Qiu H.-Y., Fu C.-C., Tang X.-W., Ruan C.-G., Wu D.-P. (2019). Incidence, risk factors, and clinical significance of Epstein–Barr virus reactivation in myelodysplastic syndrome after allogeneic haematopoietic stem cell transplantation. Ann. Hematol..

[24] Ru Y., Zhang X., Song T., Ding Y., Zhu Z., Fan Y., Xu Y., Sun A., Qiu H., Jin Z. (2020). Epstein-Barr virus reactivation after allogeneic hematopoietic stem cell transplantation: Multifactorial impact on transplant outcomes. Bone Marrow Transpl..

[25] Salas M.Q., Prem S., Remberger M., Lam W., Kim D.D.H., Michelis F.V., Al-Shaibani Z., Gerbitz A., Lipton J.H., Viswabandya A. (2020). High incidence but low mortality of EBV-reactivation and PTLD after alloHCT using ATG and PTCy for GVHD prophylaxis. Leuk. Lymphoma.

[26] Ke P., Zhang X., Liu S., Zhu Q., Ma X., Chen F., Tang X., Han Y., Fu Z., Chen S. (2021). The time-dependent effects of early-onset Epstein-Barr viremia on adult acute leukemia patients following allo-HSCT with ATG-containing MAC regimen. Ann. Hematol..

[27] Macy S., Passweg J., Medinger M. (2021). Incidence and impact of Epstein-Barr virus events in the early phase after allogeneic hematopoietic cell transplantation. Ann. Hematol..

[28] Lindsay J., Othman J., Yong M.K., Ritchie D., Chee L., Tay K., Tio S.Y., Kerridge I., Fay K., Stevenson W. (2021). Dynamics of Epstein-Barr virus on post-transplant lymphoproliferative disorders after antithymocyte globulin-conditioned allogeneic hematopoietic cell transplant. Transpl. Infect. Dis..

[29] Marzolini M.A.V., Wilson A.J., Sanchez E., Carpenter B., Chakraverty R., Hough R., Kottaridis P., Morris E.C., Thomson K.J., Peggs K.S. (2021). Natural History of Epstein-Barr Virus Replication and Viral Load Dynamics after Alemtuzumab-Based Allogeneic Stem Cell Transplantation. Transpl. Cell Ther..

[30] Chen T.-T., Lin C.-C., Lo W.-J., Hsieh C.-Y., Lien M.-Y., Lin C.-H., Lin C.-Y., Bai L.-Y., Chiu C.-F., Yeh S.-P. (2022). Antithymocyte globulin plus post-transplant cyclophosphamide combination as graft-versus-host disease prophylaxis in haploidentical peripheral blood stem cell transplantation for hematological malignancies. Int. J. Hematol..

[31] Kinzel M., Dowhan M., Kalra A., Williamson T.S., Dabas R., Jamani K., Chaudhry A., Shafey M., Jimenez-Zepeda V., Duggan P. (2022). Risk Factors for the Incidence of and the Mortality due to Post-Transplant Lymphoproliferative Disorder after Hematopoietic Cell Transplantation. Transpl. Cell Ther..

[32] Marinho-Dias J., Lobo J., Henrique R., Baldaque I., Pinho-Vaz C., Regadas L., Branca R., Campilho F., Campos A., Medeiros R. (2018). Post-transplant lymphoproliferative disorder in hematopoietic stem cell transplant patients: A single center retrospective study between 2005 and 2012. Mol. Med. Rep..

[33] Ali S., AlThubaiti S., Renzi S., Krueger J., Chiang K.Y., Naqvi A., Schechter T., Punnett A., Ali M. (2019). Hemophagocytic lymphohistiocytosis is a sign of poor outcome in pediatric Epstein-Barr virus-associated post-transplant lymphoproliferative disease after allogeneic hematopoietic stem cell transplantation. Pediatr. Transpl..

[34] Kinch A., Hallböök H., Arvidson J., Sällström K., Bondeson K., Pauksens K. (2018). Long-term outcome of Epstein-Barr virus DNAemia and PTLD with the use of preemptive rituximab following allogeneic HSCT. Leuk. Lymphoma.

[35] Neumann T., Schneidewind L., Thiele T., Pink D., Schulze M., Schmidt C., Krüger W. (2018). No indication of increased infection rates using low-dose alemtuzumab instead of anti-thymocyte globulin as graft-versus-host disease prophylaxis before allogeneic stem cell transplantation. Transpl. Infect. Dis..

[36] Tang L., Liu Z., Li T., Dong T., Wu Q., Niu T., Liu T., Ji J. (2023). Post-transplant cyclophosphamide versus anti-thymocyte globulin in allogeneic hematopoietic stem cell transplantation from unrelated donors: A systematic review and meta-analysis. Front. Oncol..

[37] Retière C., Willem C., Guillaume T., Vié H., Gautreau-Rolland L., Scotet E., Saulquin X., Gagne K., Béné M.C., Imbert B.-M. (2018). Impact on early outcomes and immune reconstitution of high-dose post-transplant cyclophosphamide vs anti-thymocyte globulin after reduced intensity conditioning peripheral blood stem cell allogeneic transplantation. Oncotarget.

[38] Massoud R., Gagelmann N., Fritzsche-Friedland U., Zeck G., Heidenreich S., Wolschke C., Ayuk F., Christopeit M., Kröger N. (2021). Comparison of immune reconstitution between anti-T-lymphocyte globulin and posttransplant cyclophosphamide as acute graft-versus-host disease prophylaxis in allogeneic myeloablative peripheral blood stem cell transplantation. Haematologica.

[39] Maeda Y. (2021). Immune reconstitution after T-cell replete HLA haploidentical hematopoietic stem cell transplantation using high-dose post-transplant cyclophosphamide. J. Clin. Exp. Hematop..

[40] Yu X.-X., Cao X.-H., Yan H., Luo X.-Y., Zhao X.-S., Sun Y.-Q., Wang Y., Xu L.-P., Zhang X.-H., Chang Y.-J. (2019). Delay expression of NKp30 on NK cells correlates with long-term mycophenolate mofetil treatment and higher EBV viremia post allogenic hematological stem cells transplantation. Clin. Immunol..

[41] Compagno F., Basso S., Panigari A., Bagnarino J., Stoppini L., Maiello A., Mina T., Zelini P., Perotti C., Baldanti F. (2020). Management of PTLD After Hematopoietic Stem Cell Transplantation: Immunological Perspectives. Front. Immunol..

[42] Alaggio R., Amador C., Anagnostopoulos I., Attygalle A.D., Araujo I.B.D.O., Berti E., Bhagat G., Borges A.M., Boyer D., Calaminici M. (2022). The 5th edition of the World Health Organization Classification of Haematolymphoid Tumours: Lymphoid Neoplasms. Leukemia.

[43] Fujimoto A., Hiramoto N., Yamasaki S., Inamoto Y., Uchida N., Maeda T., Mori T., Kanda Y., Kondo T., Shiratori S. (2019). Risk Factors and Predictive Scoring System For Post-Transplant Lymphoproliferative Disorder after Hematopoietic Stem Cell Transplantation. Biol. Blood Marrow Transplant..

[44] Lee C.-C., Hsu T.-C., Kuo C.-C., Liu M.A., Abdelfattah A.M., Chang C.-N., Yao M., Li C.-C., Wu K.-H., Chen T.-C. (2021). Validation of a Post-Transplant Lymphoproliferative Disorder Risk Prediction Score and Derivation of a New Prediction Score Using a National Bone Marrow Transplant Registry Database. Oncologist.

[45] Salmona M., Fourati S., Feghoul L., Scieux C., Thiriez A., Simon F., Resche-Rigon M., LeGoff J. (2016). Automated quantification of Epstein-Barr Virus in whole blood of hematopoietic stem cell transplant patients using the Abbott m2000 system. Diagn. Microbiol. Infect. Dis..

[46] Fryer J.F., Heath A.B., Wilkinson D.E., Minor P.D., Collaborative Study Group (2016). A collaborative study to establish the 1st WHO International Standard for Epstein-Barr virus for nucleic acid amplification techniques. Biologicals.

[47] Wareham N.E., Mocroft A., Sengeløv H., Da Cunha-Bang C., Gustafsson F., Heilmann C., Iversen M., Kirkby N.S., Rasmussen A., Sørensen S.S. (2018). The value of EBV DNA in early detection of post-transplant lymphoproliferative disorders among solid organ and hematopoietic stem cell transplant recipients. J. Cancer Res. Clin. Oncol..

[48] Rzepka M., Depka D., Gospodarek-Komkowska E., Bogiel T. (2023). Diagnostic Value of Whole-Blood and Plasma Samples in Epstein-Barr Virus Infections. Diagnostics.

[49] Lazzarotto T., Chiereghin A., Piralla A., Piccirilli G., Girello A., Campanini G., Gabrielli L., Costa C., Prete A., Bonifazi F. (2018). Cytomegalovirus and Epstein-Barr Virus DNA Kinetics in Whole Blood and Plasma of Allogeneic Hematopoietic Stem Cell Transplantation Recipients. Biol. Blood Marrow Transpl..

[50] Kanakry J.A., Hegde A.M., Durand C.M., Massie A.B., Greer A.E., Ambinder R.F., Valsamakis A. (2016). The clinical significance of EBV DNA in the plasma and peripheral blood mononuclear cells of patients with or without EBV diseases. Blood.

[51] Fink S., Tsai M.-H., Schnitzler P., Zeier M., Dreger P., Wuchter P., Bulut O.C., Behrends U., Delecluse H.-J. (2017). The Epstein-Barr virus DNA load in the peripheral blood of transplant recipients does not accurately reflect the burden of infected cells. Transpl. Int..

[52] Zhou B., Xu M., Lu S., Liu Y., Qi L., Liu T., Tian H., Chen J., Wu D., Xu Y. (2022). Clinical Outcomes of B Cell Acute Lymphoblastic Leukemia Patients Treated with Haploidentical Stem Cells Combined with Umbilical Cord Blood Transplantation. Transpl. Cell Ther..

[53] Lin R., Fan Z., Zhao K., Jiang Q., Sun J., Liu Q. (2015). Reconstitution of Epstein-Barr Virus-Specific T Lymphocytes at the Early Stage of Allogeneic Stem Cell Transplantation. Blood.

[54] Zhou X., Lu X., He J., Xu Z., Li Q., Ye P., Zhong Z., Shi W., Yan H., You Y. (2022). Clinical value of plasma and peripheral blood mononuclear cells Epstein–Barr Virus DNA dynamics on prognosis of allogeneic stem cell transplantation. Front. Cell. Infect. Microbiol..

[55] Nilsson J., Granrot I., Mattsson J., Omazic B., Uhlin M., Thunberg S. (2017). Functionality testing of stem cell grafts to predict infectious complications after allogeneic hematopoietic stem cell transplantation. Vox Sang..

[56] Stocker N., Labopin M., Boussen I., Paccoud O., Bonnin A., Malard F., Amiel C., Gozlan J., Battipaglia G., Duléry R. (2020). Pre-emptive rituximab treatment for Epstein-Barr virus reactivation after allogeneic hematopoietic stem cell transplantation is a worthwhile strategy in high-risk recipients: A comparative study for immune recovery and clinical outcomes. Bone Marrow Transpl..

[57] Kobayashi S., Sano H., Mochizuki K., Ohara Y., Takahashi N., Ohto H., Kikuta A. (2017). Pre-emptive rituximab for Epstein-Barr virus reactivation after haplo-hematopoietic stem cell transplantation. Pediatr. Int..

[58] Kim B.K., Kang H.J., Hong K.T., An H.Y., Choi J.Y., Lee J.S., Park S.S., Shin H.Y. (2019). Successful preemptive therapy with single-dose rituximab for Epstein-Barr virus infection to prevent post-transplant lymphoproliferative disease after pediatric hematopoietic stem cell transplantation. Transpl. Infect. Dis..

[59] Jain T., Kosiorek H.E., Grys T.E., Kung S.T., Shah V.S., Betcher J.A., Slack J.L., Leis J.F., Khera N., Noel P. (2019). Single dose versus multiple doses of rituximab for preemptive therapy of Epstein-Barr virus reactivation after hematopoietic cell transplantation. Leuk. Lymphoma.

[60] Solano C., Mateo E.M., Pérez A., Talaya A., Terol M.J., Albert E., Giménez E., Vinuesa V., Piñana J.L., Boluda J.C.H. (2017). Epstein-Barr virus DNA load kinetics analysis in allogeneic hematopoietic stem cell transplant recipients: Is it of any clinical usefulness?. J. Clin. Virol..

[61] Gärtner B., Preiksaitis J.K. (2010). EBV viral load detection in clinical virology. J. Clin. Virol..

[62] García-Cadenas I., Castillo N., Martino R., Barba P., Esquirol A., Novelli S., Orti G., Garrido A., Saavedra S., Moreno C. (2015). Impact of Epstein Barr virus-related complications after high-risk allo-SCT in the era of pre-emptive rituximab. Bone Marrow Transpl..

[63] Kullberg-Lindh C., Olofsson S., Brune M., Lindh M. (2008). Comparison of serum and whole blood levels of cytomegalovirus and Epstein–Barr virus DNA. Transpl. Infect. Dis..

[64] Semenova T., Lupo J., Alain S., Perrin-Confort G., Grossi L., Dimier J., Epaulard O., Morand P., Germi R. (2016). Multicenter Evaluation of Whole-Blood Epstein-Barr Viral Load Standardization Using the WHO International Standard. J. Clin. Microbiol..

[65] Patel C., Pasciolla M., Abramova R., Salerno D., Gomez-Arteaga A., Shore T.B., Orfali N., Mayer S., Hsu J., Phillips A.A. (2023). Pre-Hematopoietic Stem Cell Transplantation Rituximab for Epstein-Barr Virus and Post-Lymphoproliferative Disorder Prophylaxis in Alemtuzumab Recipients. Transpl. Cell Ther..

[66] Van Besien K., Bachier-Rodriguez L., Satlin M., Brown M.A., Gergis U., Guarneri D., Hsu J., Phillips A.A., Mayer S.A., Singh A.D. (2019). Prophylactic rituximab prevents EBV PTLD in haplo-cord transplant recipients at high risk. Leuk. Lymphoma.

[67] Kinzel M., Kalra A., Khanolkar R.A., Williamson T.S., Li N., Khan F., Puckrin R., Duggan P.R., Shafey M., Storek J. (2023). Rituximab Toxicity after Preemptive or Therapeutic Administration for Post-Transplant Lymphoproliferative Disorder. Transpl. Cell Ther..

[68] Crocchiolo R., Castagna L., El-Cheikh J., Helvig A., Fürst S., Faucher C., Vazquez A., Granata A., Coso D., Bouabdallah R. (2011). Prior rituximab administration is associated with reduced rate of acute GVHD after in vivo T-cell depleted transplantation in lymphoma patients. Exp. Hematol..

[69] Ji S.-M., Bao X.-B., Lu J., Ma X., Tao T., Sun A.-N., Wu D.-P., Xue S.-L. (2017). Protective Effect of Rituximab in Chronic Graft-Versus-Host Disease Occurrence in Allogeneic Transplant patients with Epstein Barr Virus Viremia. Indian. J. Hematol. Blood Transfus..

[70] Cutler C., Kim H.T., Bindra B., Sarantopoulos S., Ho V.T., Chen Y.-B., Rosenblatt J., McDonough S., Watanaboonyongcharoen P., Armand P. (2013). Rituximab prophylaxis prevents corticosteroid-requiring chronic GVHD after allogeneic peripheral blood stem cell transplantation: Results of a phase 2 trial. Blood.

[71] Cesaro S., Pegoraro A., Tridello G., Calore E., Pillon M., Varotto S., Abate D., Barzon L., Mengoli C., Carli M. (2010). A Prospective Study on Modulation of Immunosuppression for Epstein-Barr Virus Reactivation in Pediatric Patients Who Underwent Unrelated Hematopoietic Stem-Cell Transplantation. Transplantation.

[72] Jiang W., Clancy L.E., Avdic S., Sutrave G., Street J., Simms R., McGuire H.M., Patrick E., Chan A.S., McCaughan G. (2022). Third-party CMV- and EBV-specific T-cells for first viral reactivation after allogeneic stem cell transplant. Blood Adv..

[73] Passweg J.R., Baldomero H., Chabannon C., Basak G.W., De La Cámara R., Corbacioglu S., Dolstra H., Duarte R., Glass B., Greco R. (2021). Hematopoietic cell transplantation and cellular therapy survey of the EBMT: Monitoring of activities and trends over 30 years. Bone Marrow Transpl..

